# Botulinum Toxin for the Treatment of Postmenopausal Craniofacial Hyperhidrosis

**DOI:** 10.7759/cureus.68401

**Published:** 2024-09-01

**Authors:** Bailey Patrick, Anne-Taylor Beck, Benjamin W Casterline, Kari L Martin

**Affiliations:** 1 Department of Dermatology, University of Missouri, Columbia, USA

**Keywords:** postmenopause, adult dermatology, hyperhidrosis, craniofacial hyperhidrosis, botulinum toxin (botox) treatment

## Abstract

Postmenopausal craniofacial hyperhidrosis describes a unique subset of primary focal hyperhidrosis in menopausal woman. This condition can be challenging to treat and may require multiple treatment modalities before patients express satisfaction with the results. Current treatment options for craniofacial hyperhidrosis include oral antimuscarinic agents, topical aluminum chloride powder, and botulinum toxin injections. We present the case of a 68-year-old female with craniofacial hyperhidrosis who did not respond to topical agents and did not tolerate an oral antimuscarinic agent. The patient was successfully treated with 100 units of onabotulinum toxin A along the forehead, frontal hairline, and periauricular scalp and reported significant improvement in symptoms and quality of life as a result. We review the literature describing targeted intradermal injection of botulinum neurotoxin as an alternative to medical therapy for craniofacial hyperhidrosis.

## Introduction

Hyperhidrosis is the secretion of sweat from eccrine glands in excess of what is required for thermoregulation. The most commonly affected areas are the axillae, palms, and soles, where eccrine glands are most concentrated, although any body location can be affected. Primary hyperhidrosis is idiopathic and represents most cases, whereas secondary hyperhidrosis has an etiology such as medication adverse effects or endocrine conditions. Current first-line treatment options for localized hyperhidrosis include topical antiperspirants like aluminum chloride and topical or systemic anticholinergic agents such as glycopyrrolate [1]. 

Injection of botulinum toxin into affected areas can be effective for patients who do not respond to these therapies. Botulinum toxin acts by cleaving SNAP-25 protein on the presynaptic membrane of the nerve terminus. This action prevents acetylcholine release into the synapse, thereby inhibiting sympathetic stimulation of eccrine glands. Botulinum toxin has been demonstrated to significantly reduce sweating in the axillary region [2]. Botulinum toxin also has demonstrated efficacy and improved patient quality of life in the palmoplantar areas [3]. Other areas affected by primary hyperhidrosis have received less attention.

Craniofacial hyperhidrosis has been successfully treated with oral antimuscarinic agents, topical aluminum chloride, and botulinum toxin injections. The treatment of craniofacial hyperhidrosis has been less extensively studied than axillary hyperhidrosis, but a review of the literature shows evidence to support botulinum toxin use and provides guidance about dosing and administration.

## Case presentation

A 68-year-old female patient presented to the dermatology clinic with a concern about excessive sweating of the forehead and frontal scalp after minimal activity in warm weather. The patient had no precipitating factors and had not recently started any new medications. When the patient first was concerned about excessive sweating, she was taking sertraline, metaxalone, amitriptyline, atenolol, hydrochlorothiazide, losartan, and simvastatin. The symptoms of hyperhidrosis occurred before initiating sertraline and persisted after discontinuation of sertraline and amitriptyline.

**Figure 1:**
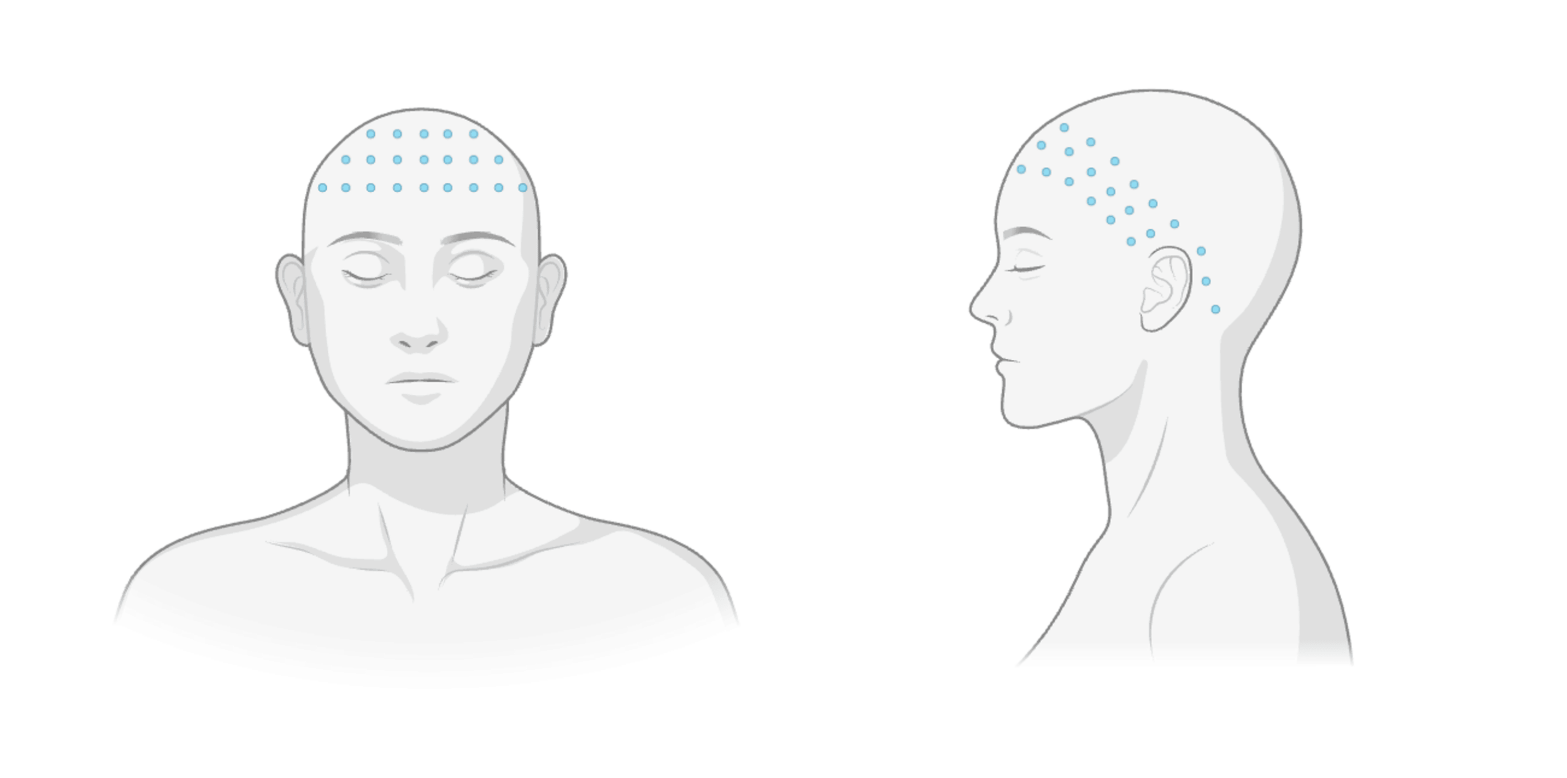
Injection sites for the patient around the upper forehead and frontal hairline, temporal hairline, and around the auricle to the mastoid. Created with BioRender from the injection sites of the patient at the office visit.

For hyperhidrosis management, this patient was first prescribed glycopyrrolate 1 mg three times daily but was unable to tolerate it due to eye dryness. The patient then tried topical aluminum chloride on the scalp but reported minimal effectiveness. At the next visit, she was treated with 100 units of onabotulinumtoxinA along the superior forehead and occipital hairline (Figure 1). The patient noted significantly improved sweating over the summer and improved quality of life from the treatment. She returned for four follow-up treatments that were 11 months apart on average.

## Discussion

We report a case of primary craniofacial hyperhidrosis treated successfully with botulinum toxin monotherapy. The treatment of craniofacial hyperhidrosis has been less extensively studied than axillary hyperhidrosis, but multiple pieces of literature have shown evidence to support botulinum toxin use (Table 1). 

**Table 1:** Current literature on the treatment of craniofacial hyperhidrosis with botulinum toxin Citation: [4-8]

Study	Design	Patient population	Patient number	Medication	Injection sites	Treatment efficacy
Cabreus, 2019 (6)	Randomized control trial	Postmenopausal women with craniofacial hyperhidrosis, average age 67.4	8	2250 units of botulinum toxin type B	Every 15 mm across the frontal, temporal, and occipital scalp, forehead and glabella, infraocular and perioral areas; 2.5–7.5 units/injection	Improved quality of life for patients who received botulinum toxin injection three weeks after injection
Eustace, 2018 (4)	Case series	Postmenopausal women with craniofacial hyperhidrosis, average age 63.9	11	100 units of botulinum toxin type A	"Typically the hairline and nape of the neck"; 40 injections of 2.5 units	5.3 months mean duration of effect
Hanna-Bashara, 2013 (5)	Case report	Postmenopausal woman with craniofacial hyperhidrosis, age 49.	1	100 units of botulinum toxin type A and oral glycopyrrolate	Hairline and forehead	The patient achieved partial control of symptoms with oral glycopyrrolate but full control with oral and injection combination.
Karlqist, 2013 (7)	Prospective open study	Men and women of any age with craniofacial hyperhidrosis, average age of 44	38	Botulinum toxin type B, total dose 110 U to 2300 U depending on the patient’s area of concern.	Every 15 mm across the frontal, temporal, and occipital scalp, forehead and glabella, infraocular and perioral areas; 5 units/injection	Five months median time to retreatment
Komericki, 2012 (8)	Case report	Male with hyperhidrosis of face and scalp, age 52	1	100 units of botulinum toxin type A	300 injection sites across perioral, nasolabial, forehead, and scalp	Treatments repeated every five to six months

Postmenopausal craniofacial hyperhidrosis represents a unique subset of hyperhidrosis patients. This type of hyperhidrosis can affect any menopausal woman regardless of if they are taking estrogen or progesterone. A 2018 case series detailed the demographics and treatment of 20 women with postmenopausal craniofacial hyperhidrosis [4]. All patients in this study reported that their condition significantly negatively impacted their quality of life. All 20 women in the study had tried oral anticholinergic medications, including glycopyrrolate, but only 30% of the women had a complete response to oral anticholinergics. Eleven of the patients in the group were eventually treated with 100 units of botulinum toxin type A injections spaced over the areas where patients endorsed sweating, usually the hairline and nape. The majority of those treated with the botulinum toxin type A rated the treatment as “life-changing.” The mean duration of effect for the craniofacial botulinum toxin injections was 5.33 months in this study.

Multiple case reports have demonstrated successful treatment of craniofacial hyperhidrosis using botulinum toxin injections. A 2013 case report discussed a 49-year-old woman with postmenopausal craniofacial hyperhidrosis of the scalp who was successfully treated with a combination of oral glycopyrrolate and 100 units of botulinum toxin type A [5]. The patient was first treated with oral glycopyrrolate and achieved partial control of symptoms. The patient achieved full control of symptoms with the botulinum toxin injections. 

Preliminary data from a randomized clinical trial in Sweden indicate that botulinum toxin type B for postmenopausal craniofacial hyperhidrosis significantly reduces measured sweating and improves quality of life metrics [6]. Eight patients were part of a randomized control trial for postmenopausal women with hyperhidrosis and were randomized to placebo injections or 2250 units of botulinum toxin type B. A quality-of-life survey performed at baseline and three weeks after treatment that demonstrated the botulinum toxin type B significantly improved the quality of life for patients injected with it.

## Conclusions

Botulinum toxin is an effective treatment for postmenopausal craniofacial hyperhidrosis that is refractory to oral antimuscarinics and topical agents. In our patient, 100 units of onabotulinumtoxinA distributed evenly across the forehead, frontal hairline, and periauricular scalp were effective for symptomatic relief for up to five months. The literature supports other craniofacial sites for treatment. Treatment should be tailored to the patient’s symptoms.
